# Computational approaches in target identification and drug discovery

**DOI:** 10.1016/j.csbj.2016.04.004

**Published:** 2016-05-07

**Authors:** Theodora Katsila, Georgios A. Spyroulias, George P. Patrinos, Minos-Timotheos Matsoukas

**Affiliations:** a University of Patras, School of Health Sciences, Department of Pharmacy, University Campus, Rion, Patras, Greece; b Department of Pathology, College of Medicine and Health Sciences, United Arab Emirates University, Al-Ain, United Arab Emirates

**Keywords:** Data integration, Information technologies, Target identification, Computer-aided drug discovery

## Abstract

In the big data era, voluminous datasets are routinely acquired, stored and analyzed with the aim to inform biomedical discoveries and validate hypotheses. No doubt, data volume and diversity have dramatically increased by the advent of new technologies and open data initiatives. Big data are used across the whole drug discovery pipeline from target identification and mechanism of action to identification of novel leads and drug candidates. Such methods are depicted and discussed, with the aim to provide a general view of computational tools and databases available. We feel that big data leveraging needs to be cost-effective and focus on personalized medicine. For this, we propose the interplay of information technologies and (chemo)informatic tools on the basis of their synergy.

## 1 Introduction

Current trends in drug discovery focus on disease mechanisms and their understanding, followed by target identification and lead compound discovery. In the era of personalized medicine and better-informed cost-effective public health outcomes, a system of personalized medicine that is based on molecular states (and changes, from DNA to RNA to protein) have become fundamental in drug discovery [1], [2]. To build such a system, the molecular characterization of disease is necessary, while environmental influences and the gut microbiome needs to be also considered [3], [4]. At the same time, regulatory requirements of safety are increasing [5].

To address the above-mentioned interplay in high-throughput formats, we feel that information technologies and chemoinformatic tools need to be employed on the basis of a synergy that even extends to artificial and human intelligence interplay - humans can detect patterns, which computer algorithms may fail to do so, whereas data-intensive and cognitively complex settings and processes limit human ability [6]. We propose that this synergy will (i) facilitate collaborative data analysis and (ii) guide sense- and decision-making towards rapid and efficient data output. Big and diverse data demand strict filtering and thorough analysis and interpretation. At the same time, biomedicine scientists need to efficiently and effectively collaborate and make decisions. For this, large-scale volumes of complex multi-faceted data need to be meaningfully assembled, mined and analyzed [7]. In such a context, reliable target identification and validation in cooperation with drug discovery methods will pave the way to more efficient computer aided drug discovery. Moreover, new network-based computational models and systems biology integrate omics databases and optimize combinational regimens of drug development.

## 2 Target identification

Chemoinformatic tools present a tremendous potential to advance *in silico* drug design and discovery, as they serve the integration of information in several levels to enhance the reliability of data outcomes. To name a few, chemical structure similarity searching [8], data mining/machine learning [9], panel docking [10], and bioactivity spectra based algorithms [11] have been routinely and successfully implemented [12], [13]. Some examples are the ligand-based interaction fingerprint (LIFt) approach [14] in predicting potential targets for small-molecule drugs using physics-based docking and sampling methods and the protein ligand interaction fingerprints (PLIF) method [15] for summarizing interactions between ligands and proteins using a fingerprint scheme. In both cases, compounds were identified for the p38α MAP kinase and GPR17, respectively (Table 1).

Target identification can also be studied through network-based drug discovery, a field integrating different levels of information in drug-protein and protein-disease networks. This approach involves a highly collaborative scheme between databases and correlations across genomics, transcriptomics, proteomics, metabolomics, microbiome, pharmacogenomics, which highly depends on the development of relevant computational and systems biology tools for such data interpretation [16], [17]. Such approaches, for example relating pharmacological and genomic spaces can be used to develop computational frameworks for drug target identification [18]. Another recent network-based application was the integration of large-scale structural genomics and disease association studies, to generate three-dimensional human interactome, that resulted in the identification of candidate genes for unknown disease-to-gene associations with proposed molecular mechanisms [19].

To facilitate gaining in-depth knowledge of disease mechanisms and/or phenotypes information technologies are greatly needed today more than ever [20]. Indeed, the study of disease mechanisms and/or phenotypes has turned from investigating a particular gene or protein into the analysis of entire sets of biomolecules [21]. The advent of omics technologies further complicates storing, visualizing and analyzing voluminous biological data. For this, information technologies provide the means towards extensive data processing and interpretation. Tools such as the human metabolome database [22] and MetaboAnalyst [23] support integrative omics pathway analysis. The human metabolome database contains metabolite entries linked with chemical, clinical, and molecular biology data, that can assist applications in metabolomics, clinical chemistry and biomarker discovery. Metaboanalyst is a web-based analytical pipeline for high-throughput metabolomics studies, which offers a variety of procedures for metabolomic data processing and integrates biomarker and pathway analysis. MAGENTA (http://www.broadinstitute.org/mpg/magenta/) and Ingenuity (http://www.ingenuity.com/) users can further exploit several curated biological pathways. Databases play a key role and no doubt, an extremely rich repertoire is available today (Table 2). When kinome is of interest, a computational platform ReKINect has been recently reported to identify network-attacking mutations and validated with the interpretation of exomes and quantitative proteomes of ovarian cancer cell lines and the global cancer genome repository [24]. Another useful approach helping to identify functional connections between diseases, genes and drugs is the Connectivity Map [25]. Connectivity Map is a collection of genome-wide transcriptional expression data from cultured human cells treated with bioactive small molecules and simple pattern-matching algorithms that together enable the discovery of functional connections between drugs, genes and diseases through the transitory feature of common gene-expression changes [26]. Other computational methods have been also applied to reconstruct biological networks and extract information from them, such as Bayesian [27] and Boolean networks [28] and graph based models [29].

Furthermore, applications and web services, enable sharing of data and resources for visualization and analysis purposes. The Biological General Repository for Interaction Datasets (BioGRID) [30] is an interaction repository with compiled biological data freely available in standardized formats, linked with software platforms for visualization of complex interaction networks such as Osprey [31] and Cytoscape [32]. BioMart, is a community-driven project, which call for scientists to share data and provides free software and data services to the scientific community in order to facilitate scientific collaborations and the scientific discovery process [33]. Oncomine, is a cancer microarray database and web-based data-mining platform aimed at facilitating discovery from genome-wide expression analyses, providing with query and visualization tools for selected or multiple genes across all analyses [34]. The online Cancer-Related Analysis of Variants Toolkit (CRAVAT) can assist the high-throughput assessment and prioritization of genes and missense alterations important for cancer tumorigenesis, by providing predictive scores for germline variants, somatic mutations and relative gene importance [35]. The Sorting Intolerant from Tolerant (SIFT) algorithm predicts the effect of coding variants on protein function through a web server. It provides users with predictions on their variants and is widely used for characterizing missense variations [36]. PROVEAN (Protein Variation Effect Analyzer) is a software tool which predicts whether an amino acid substitution or indel has an impact on the biological function of a protein [37] and GenePattern provides with analytical tools for the analysis of gene expression, sequence variation and network analysis. MetaMapR, an open source software integrates enzymatic transformations with metabolite structural similarity, mass spectral similarity and empirical associations to generate connected metabolic networks [38]. Protein and DNA visualization software such as VMD [39] and Chimera [40] are widely used in the 3D analysis of biomolecules and drug interactions. Several networks (caBIG, http://cabig.cancer.gov; BIRN, http://www.nbirn.net) and projects (Genotype-Tissue Expression Project [41]; RD-Connect [42]) have been initiated towards data exchange for target identification.

Another important field in information technologies is the semantics field, which could give insights to associations between heterogeneous data of diseases and drug targets. Such network-based computational approaches have gained popularity recently, proposing novel therapeutic targets and deciphering disease mechanisms. However, little effort has been devoted to investigating associations among drugs, diseases, and genes in an integrative manner. In such a study, Zhang et al., constructed an association network by extracting pair-wise associations between diseases, drugs and genes in Semantic MEDLINE and applied a network-based approach to mine the local network structure [43]. This could result in the formulization of novel research hypotheses, which is critical for translational medicine research and personalized medicine.

## 3 Target validation

Target validation is a time-consuming and costly process that demonstrates relevance – is the identified target of relevance to a particular biological pathway, molecular process or disease? We agree that target validation efficiency can be greatly improved when combined to strict data filtering and statistics, as high throughput screening sheds light to cellular responses in disease models of interest. Network validation can be performed by comparing the network of interest to 100 random networks generated using random shuffling of the graph with degrees preserved as implemented in the Randomized network plugin in Cytoscape2.6.3 [32]. Gene function and/or gene regulatory networks can be validated *via* genome-wide approaches [44] and functional screens, such as RNAi and CRISPR-Cas9 [45]. Inter-individual variability upon drug administration/ intervention can be tracked and analyzed recently, as electronic medical records and clinical trial data become available. In addition to the molecular and clinical data, free-text data presented in literature are also useful in drug discovery *via* extensive data mining processes [46].

## 4 Computer-aided drug design

Once a target has been identified, there are several *in silico* tools to initiate a drug design process. The use of these methods depends on the nature of the target and the available information on the system. In the past decade, computer aided drug design (CADD) has offered valuable tools in the identification of compounds, minimizing the risk of later rejection of lead compounds. Even though high throughput screening (HTS) usually offers several hit compounds, success rates are often very low and many of the identified compounds are later rejected due to their physicochemical properties. CADD plays a significant role in high success rates of hit compound identification [47], as well as the prioritization of HTS active compounds. One of many examples of the importance of CADD compared to HTS, was the identification of inhibitors against the transforming growth factor-b 1 receptor kinase. While the HTS for compound identification at Eli Lilly resulted in a potent lead compound [48], at the same time, a fully computational approach by Biogen Idec [49], resulted in the identification of 87 hits, the best one being Eli Lilly's initial lead compound [50]. In this case, a fully computational work was able to produce the same result as a wet lab approach, which traditionally is more costly and time consuming.

**Fig. 1:**
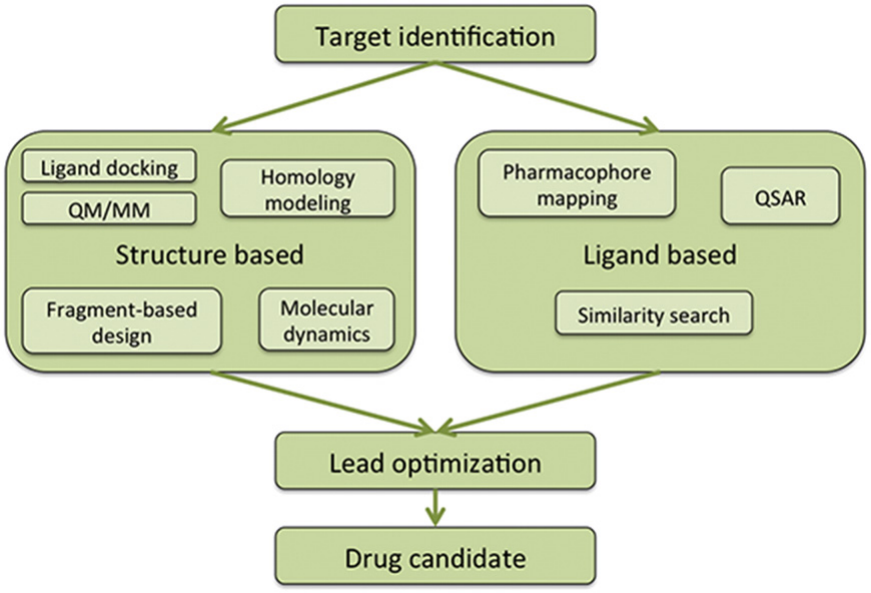
General computer aided techniques for drug design. The usage largely relies on the available structural information on the drug target to be assessed. No structural information leads to ligand based drug design methods, where known active compounds are used for the discovery of similar, more potent drug candidates.

There are generally two distinct methods for computational drug design, *structure based* and *ligand based* (Fig. 1). These depend on the available information on the identified target. Most of them are analyzed in detail elsewhere [51], [52], however the scope of this mini-review is to highlight and review the most commonly used. When there is no information on the structure of the target, computational methods for new molecules are based on information of known active or inactive compounds against it. This is the ligand based CADD approach, where tools such as ligand chemical similarity or pharmacophore mapping can be very useful. Generally, the most commonly used methods in hit compound identification rely on virtual screening techniques on the targets' binding site. These methods mostly rely on docking large libraries of small molecules such as ZINC [53], or chemical information on known compounds such as Pubchem [54] using docking or pharmacophore modeling tools. The use of such libraries, however, is expensive from a computational perspective. If no adequate computational resources are available, cascade virtual screening protocols are applied in a way that databases are filtered based on physicochemical or other properties of the compounds to avoid using databases as a whole [55], [56].

There are studies in which ligand similarity-based virtual screening and structure based virtual screening results for the same targets and compound sets have been compared. For example, in a study comparing these two methods in several drug targets (CDK2, COX2, estrogen receptor, neuraminidase, HIV-1 protease, p38 MAP kinase and thrombin), the ligand-based virtual screening methods performed better in most of the cases [57]. In another example, the comparison between ligand-based and structure-based methods (vROCS and FRED) demonstrated that the ligand based method performed in a better predictive matter [58].

The combination of ligand and structure based molecular modeling methods, however, has become a common approach in virtual screening through sequential, parallel or hybrid approaches [59]. For example, hybrid protein–ligand pharmacophore methods have been successfully applied in virtual screening [60] as well as ligand profiling studies [61]. These methods can also be integrated in network-based approaches towards drug discovery [16], [62]. Furthermore, other combinatorial tools can be implemented for the aim of multi-targeted drug design [63]. Such drugs, produced with one single chemical or with a composition of several chemicals, should be able to target the characteristic pathological network of a disease. A characteristic example, combination of dasatinib, a ABL/T315I inhibitor in combination with imatinib, a tyrosine-kinase inhibitor are proposed to treat chronic myelogenous leukemia (CML) by targeting BCR–ABL fusion proteins [64].

### 4.1 Ligand-based CADD

Ligand based CADD methods take advantage of information of small molecules interacting with the target in question in order to identify new, more potent compounds. This information includes binding affinities, chemical structure, physicochemical properties *etc.* These methods are considered in some cases more successful than structure based techniques [65].

One ligand-based approach is the selection of new compounds based on chemical similarity of known active ones. This can be done using several fingerprint methods, which allow the representation of a molecule in a way that can be effectively compared against other molecules. These methods rely on the chemical information of compounds, giving a highly qualitative approach in the search of new more potent ligands [66]. A representative example of identifying T-type calcium channel blockers, which are implicated in epilepsy and neuropathic pain [67] is the work reported by Ijjaali and co-workers [68]. In this work, a ligand based virtual screening was made on a two million compound database using ChemAxon's PF and CCG's GpiDAPH3 fingerprints to test 38 molecules for their ability to affect the functional activity of recombinant human Ca_V_3.2 (Table 1). Sixteen out of the 38 molecules were active hits as they showed more than 50% blockade of the Ca_V_3.2 mediated T-type current.

Another significant approach is the quantitative structure–activity relationship (QSAR), where a QSAR model is able to describe a correlation between structures of a set of molecules and their target response [69]. The general QSAR workflow consists of the gathering of a set of active and inactive molecules against a target and production of the descriptors describing their structural and physicochemical properties. The model can then be used to correlate these descriptors and the experimental activity, resulting in a predictive tool for new molecular entities [69]. QSAR algorithms are continuously evolving, involving the implementation of several 2D and 3D descriptors, which can be structural or physicochemical (*e.g.* molecular weight, volume, rotatable bonds, interatomic distances, atom types, molecular walk counts, electronegativity, atom distribution, aromaticity, solvation properties) and can be described on multiple levels of increasing complexity. A recent work, highlighted the success of such a method, being able to identify hit compounds that act as allosteric modulators of mGlu5. mGlu5 is a well established pharmaceutical target against anxiety, Parkinson's disease, and schizophrenia [70]. Building a QSAR model, with information from a previous HTS screen on mGlu5 [71] Mueller and co-workers were able to identify 27 active compounds that modulates the signaling of the protein [72]. The overall success of the QSAR model was a 3.6% hit rate [72] compared to the 0.2% hit rate of the HTS [71].

Another field gaining ground in the computational drug discovey setting is proteochemometrics and polypharmacology modeling [73]. Proteochemometrics (PCM) modeling combines both ligand information and target information within a single predictive model in order to predict an output variable of interest [74], [75]. Ligand information of the system is accompanied by information for its biological effect. Merging data from ligand and target sources into the frame of a single machine learning model allows the prediction of the most suitable pharmacological treatment for a given genotype (personalized medicine), which ligand-only and protein-only approaches are not able to perform [74], [75]. Using PCM methods, Frimurer at al managed to identify ~ 60 ligands for the prostaglandin D2 receptor 2 (CRTH2), after screening of a library of 1.2 million compounds [76].

### 4.2 Structure-based CADD

3D information on proteins and DNA started being used for drug design almost three decades ago. The protein databank (PDB) is the largest depository of biomolecule structure information determined mostly by X-ray crystallography and NMR techniques. In 1998, 2058 structures had been deposited in the protein data bank. Since then, each year there has been a ~ 7.5% increase in depositions, resulting in a total of 105,465 structures in 2014. The use of this abundant structural information has been the cornerstone for structure based drug design for the past years in academia, as well as the pharmaceutical industry.

Proteins, by nature are dynamic macromolecules. Thus, a structural snapshot is not enough to study a protein's interaction with a small molecule, or even identify its binding site. One of the most important aspects of studying the behavior of proteins is called molecular dynamics (MD) simulations. Based on Newtonian mechanics and using force fields such as Amber [77] or CHARMm [78], molecular dynamics simulations can calculate a trajectory of conformations as a function of time. In MD simulations, chemical bonds and atomic angles are modeled using simple virtual springs, and dihedral angles are modeled using a sinusoidal function. Non-bonded forces occur as van der Waals interactions, as the Lennard-Jones 6–12 potential and Coulomb's law are used to calculate hydrophobic and electrostatic interactions. These simulations, coupled with experimental data have a significant impact on the drug discovery field.

Traditionally these calculations have been performed in cpu clusters with software able to parallelize the processes of simulation complex systems. In the recent years, calculations required for these simulations have been developed to be performed by video-game and computer-graphics applications. Eventually the graphics-processing-units (GPUs) designed to speed up video games have began to be used to speed up molecular dynamics simulations as well, usually by an order of magnitude [79], [80].

Several MD applications are used for free energy calculations in order to correlate experimental binding affinities of small molecules to a protein with calculated, such as molecular mechanics Poisson–Boltzmann surface area (MM/PBSA) [81], linear interaction energy (LIE) [82], and free energy perturbation methods (FEP) [83]. They can then be used for the prediction of binding affinities *in silico.* Some examples of the use of such methods are the reproduction of binding free energies TIBO derivatives to HIV-1 RT and prediction of the binding mode of efavirenz to HIV-1 RT by MM-PBSA [84], the relative binding free energy calculations of the interaction of biotin and its analogs with streptavidin using FEP approaches [85] and LIE models for predicting the binding mode of β-secretase (BACE) inhibitors [86].

**Fig. 2:**
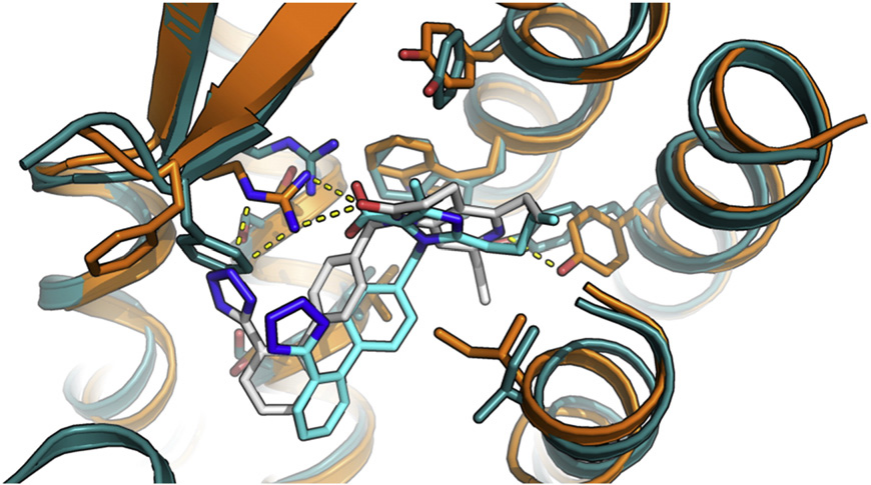
Predicted homology model of the angiotensin II type 1 receptor (deep blue) with compound EXP3174 docked (cyan) superimposed with the crystal structure of AT1 (orange) co-crystallized with ZD7155 (white). Hydrogen bonds are depicted in yellow dashed lines.

In some cases, the structure of a protein target for a drug design project may not be yet solved. In this case, there are predictive tools for building comparative models. Comparative modeling is used to predict the structure of a protein based on a structural template with a similar sequence, with the general view that proteins with similar sequences have similar structures. Homology modeling is the most used computational technique towards this goal and is used to construct a protein model after identification of a structural template protein with similar sequence, alignment of their sequences, using coordinates of aligned regions, prediction of missing atom coordinates of the target, model building and refinement. Some of the most widely used software for homology modeling are MODELER [87] and SWISS-MODEL [88]. There are several examples of the use of homology modeling in drug design, one of which the model of chemo-attractant receptor OXE-R based on the crystal structure of CXCR4 [89] as a template. Consecutively, using virtual screening techniques a small-molecule modulator Gue1654 was identified, which inhibits a specific GPCR signaling pathway [90]. In another example, the binding mode of antihypertensive drugs to the angiotensin II receptor type 1 (AT1) was predicted [91]. A homology model of the receptor and analysis of binding mode of active compounds using MD and pharmacophore modeling was later validated when the crystal structure was determined (Fig. 2) [92].

Docking methods are used to predict the preferred orientation of one molecule to a protein when bound to each other to form a stable complex. Depending on the method, there are different considerations of the flexibility of either the ligand or the protein during the docking process [93]. The most commonly used method considers the ligand flexible, while the protein docking site is held rigid, usually pre-treated with molecular dynamics force fields. Several software packages are available for docking, such as Gold [94], Autodock [95], AutoDock Vina [96], DOCK [97], GLIDE [98], SURFLEX [99] and others. The docking score is an evaluation of the energetic affinity of the complex, calculated by scoring functions. These scoring functions can be molecular mechanics-based, empirical, knowledge-based, or consensus-based functions. For example, DOCK uses the AMBER [77] force field for evaluating the energetics of binding, while SURFLEX uses an empirical function. Consensus scoring is a method whereby the binding affinities of compounds for a particular target are predicted by using more than one scoring algorithm and is a frequently studied method [100]. In such a study, Tuchinardi et al. evaluated the consensus docking and scoring of several different algorithms along 83 ligand-receptor X-ray structures [101].

Another widely used method for evaluating the probability of molecule binding to protein binding sites is pharmacophore modeling. A pharmacophore is the ensemble of steric and electronic features that is necessary to ensure the optimal supramolecular interactions with a specific biological target structure. By mapping the interaction of an active compound bound to its target protein, a pharmacophore can represent the geometrical and chemical properties using pharmacophore features, which include hydrogen bond acceptors and donors, basic and acidic groups, partial charge, aliphatic and aromatic hydrophobic moieties. This representation can be used for virtual screening projects, in order to identify potential binders based on this interaction. Several software packages have been developed towards pharmacophore modeling, Ligandscout [102] and The Pocket v.2 [103] use algorithms in protein-ligand complex data to map interactions between ligand and target.

**Fig. 3:**
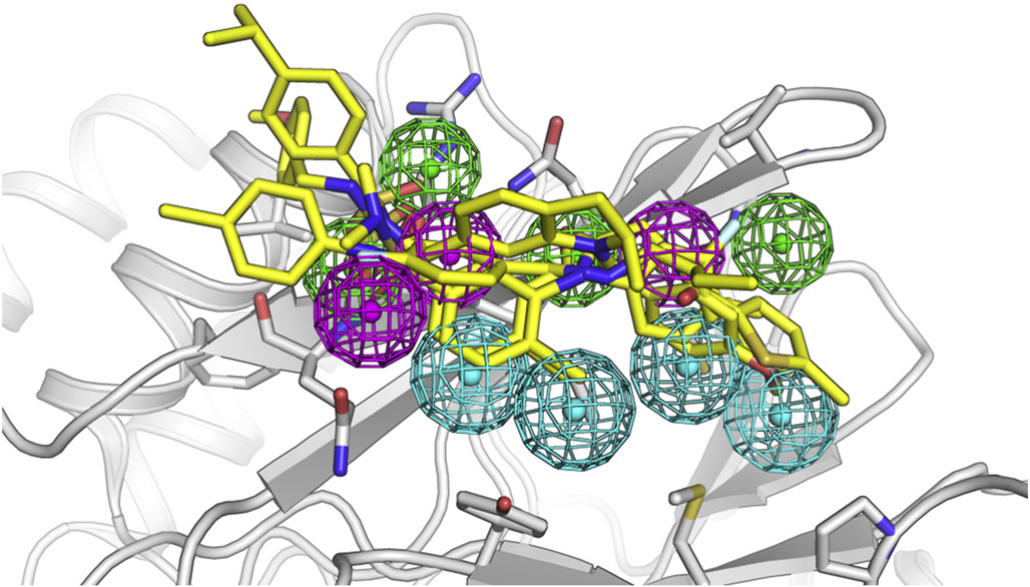
Four pharmacophore model hit compounds (shown in yellow stick representation), which were identified through a virtual screening lead compound identification process to target Calcineurin (in white ribbon and stick representation). The pharmacophore model is shown in colored spheres (cyan for hydrophobic, magenta for hydrogen bond donor and green for hydrogen bond acceptor features).

Docking and pharmacophore modeling have been widely used in virtual screening studies to identify novel compounds against drug targets. There are several successful examples of hit compounds that later on proceeded to a hit to lead process. Human Pim-1 kinase, a highly conserved serine–threonine kinase is a valuable anticancer drug target. In a virtual screening study using docking methods and a database of 700,000 commercially available compounds, four compounds were identified, having affinity in the micromolar range [104]. In another study, using a docking screening approach to identify novel dual kinase (EGFR)/bromodomain (BRD4) inhibitors from six million commercially available small molecules, Allen and co-workers selected and tested 24 compounds [105]. The result was the identification of several novel BRD4 binders and one novel dual EGFR-BRD4 inhibitor (2870), a first in class compound that could target multiple cancer promoting pathways. The use of pharmacophore modeling in high throughput virtual screening has also proven valuable in identifying novel active compounds. In a study to identify Calcineurin inhibitors that could be further developed into novel immunosuppressant agents, a fifteen million database of purchasable compounds from ZINC was screened on a pharmacophore model mapping a protein–protein interaction. Out of the 32 compounds tested, seven showed micromolar affinity, with four of them having the ability to inhibit the expression of nuclear factor of activated T cells (NFATc) dependent genes, cytokine production, and cell proliferation, suggesting that these may have therapeutic potential as immunosuppressive agents (Fig. 3) [106].

Once a computational hit identification method has produced compounds of usually low affinity, fragment-based drug discovery (FBDD) is utilized for finding lead compounds as part of the drug discovery process. Commercial databases of compounds have the limitation of representing only a small amount of the available chemical space. It has been estimated that the Lipinski virtual chemical space might contain as many as 10^60^ compounds [107]. This is where FBDD comes to enrich hit compounds and produce more potent lead compounds [108]. The two commonly used approaches for the optimization of fragment hits into lead-like compounds are Fragment growing and Fragment linking. The first is the addition of functional groups to the active fragment core in order to optimize interactions with the binding site, while the second is a less commonly used method, which links fragments that bind in adjacent sites of a target protein to turn low affinity fragments into high affinity leads. Successful examples of hit optimization are well documented, such as the discovery of Beta-site amyloid precursor protein cleaving enzyme 1 (BACE1) by Amgen towards inhibitors against Alzheimer's disease [109]. Another example is the fragment based discovery of inhibitors against the phosphatidylinositol-3 kinases (PI3Ks) [110] which are involved in cancer, rheumatoid arthritis, cardiovascular disease and respiratory disease.

## 5 Summary and outlook

Computational methods have provided a powerful toolbox for target identification, discovery and optimization of drug candidate molecules. Information technologies coupled to statistics and chemoinformatic tools shed light to disease mechanisms and phenotypes revealing potential drug targets to be further validated by high throughput screening technologies.

Consecutively, multiple methods allow for the prediction and characterization of binding sites, studying the dynamic nature of drug targets, identifying new active molecular entities and their optimization. Nowadays, large databases of readily commercially available compounds and ligand chemical space exploration offer drug discovery scientists with enormous data to handle. Different methods, based on readily available information on the biological system under study are evolving to assist the manipulation and handling of this data. Moreover, integration of ‘-omics’ technologies and databases may facilitate the identification of novel drug targets or the design of network-based multi-target drugs. Structure and ligand based methods are the most commonly used in the drug discovery field, however, emerging combinatorial techniques such as proteochemometrics are emerging.

All the computational methods mentioned in this review, either towards target identification, either towards novel ligand discovery continue to evolve and their synergy is what we envisage that will facilitate cost-effective and reliable outcomes in an era of big data demands.

## Figures and Tables

**Table 1:** Drug targets and computational methods used for compound identification and interaction prediction.

Drug target	Computational approach	Reference
p38α MAP kinase	Ligand-based interaction fingerprint (LIFt)	[14]
GPR17	Protein ligand interaction fingerprints (PLIF) method	[15]
Transforming growth factor-b 1 receptor kinase (TGFβ)	Shape-based screening (CatShape, Catalyst)	[49]
T-type calcium channel (CaV)	Bidimensional pharmacophoric fingerprints (ChemAxon and CCG's GpiDAPH3 fingerprints)	[68]
Metabotropic glutamate receptor 5 (mGlu5)	Artificial neural network (ANN) quantitative structure–activity relationship (QSAR)	[72]
prostaglandin D2 receptor 2 (CRTH2)	Proteochemometrics modeling (PCM)	[76]
HUMAN immunodeficiency virus 1 reverse transcriptase (HIV-1 RT)	Molecular mechanics energies combined with the Poisson-Boltzmann surface area (MM-PBS)	[84]
Biotin	Molecular dynamics/free energy perturbation (FEP)	[85]
β-Secretase (BACE)	Linear interaction energy (LIE)	[86]
Chemo-attractant receptor (OXE-R)	Docking virtual screening (PyPx and AutoDock Vina)	[90]
Angiotensin II receptor type 1 (AT1)	Ligand based pharmacophore modeling (Catalyst)	[91]
Pim-1 kinase	Docking virtual screening (Glide)	[104]
Epidermal growth factor receptor (EGFR)/Bromodomain-containing protein 4 (BRD4)	Docking virtual screening (Glide)	[105]
Calcineurin	Structure based pharmacophore virtual screening (Discovery Studio)	[106]

**Table 2:** Web-accessible databases for drug target identification.

Utility	Url
Human metabolome data	http://www.hmdb.ca
*In silico* target identification	http://www.dddc.ac.cn/pdtd/
Pathway analysis	http://www.genome.jp/kegg/
	http://www.geneontology.org
	http://www.reactome.org
	http://www.pantherdb.org
	http://www.biocarta.com
	http://www.ingenuity.com/
Chemogenomic data	http://www.ebi.ac.uk/chembldb
	http://pubchem.ncbi.nlm.nih.gov
Drug target database	http://www.drugbank.ca
Protein data bank	http://www.pdb.org
Disease specific target database	http://thomsonreuters.com/metacore
Pharmacogenomic data	http://www.pharmgkb.org
Multi-level drug data	http://r2d2drug.org/DMC.aspx
Comparative toxicogenomic database	http://ctdbase.org
Target-toxin database	http://www.t3db.org
Protein expression information	http://www.proteinatlas.org
Therapeutics target database	http://bidd.nus.edu.sg/group/cjttd/
